# Distilling Knowledge in Gastroenterology: An Artificial Intelligence System for Assisting Colonoscopy and Pathology Report Review

**DOI:** 10.1016/j.mcpdig.2026.100372

**Published:** 2026-05-16

**Authors:** Brayden Mau, Sushil Kumar Garg, Sarah B. Harper, Rahul Gomes, Alexandra Rolli, Rajeev Chaudhry

**Affiliations:** a Department of Computer Science, University of Wisconsin-Eau Claire, Eau Claire, WI; b Division of Gastroenterology and Hepatology, Mayo Clinic Health System, Eau Claire, WI; c AI and Bioinformatics, Mayo Clinic Health System, Rochester, MN; d Division of Community Internal Medicine, Mayo Clinic College of Medicine and Science, Rochester, MN

## Abstract

**Objective:**

To develop an efficient and domain-adapted system to process colonoscopy and pathology reports using knowledge distillation techniques.

**Patients and Methods:**

We implemented a knowledge distillation framework to create a smaller, domain-specific large language models-based natural language processing model for summarizing and extracting key information from clinical reports. The model was trained on a dataset consisting of 5500 colonoscopy reports and 7000 pathology reports taken from January 1, 2024, to June 30, 2024. Performance was evaluated against ground truth polyp categories derived from pathology report diagnoses.

**Results:**

The distilled model reported high domain-specific performance, achieving 95.2% accuracy (95% CI, 93.9%-96.5%), 0.95 precision, and a 31.5% improvement in inference speed relative to the teacher model. Despite being substantially smaller than the teacher model, it maintained strong capability in polyp category identification from key clinical factors, including polyp number, size, histology, and location across colonoscopy and pathology reports. Domain clinicians reported high agreement with model outputs across all 6 evaluated clinical questions, confirming its reliability for supporting follow-up recommendation workflows.

**Conclusion:**

This work presents a step toward making domain-specific natural language processing models for gastroenterology more efficient and scalable. By leveraging knowledge distillation, we report the potential for creating more practical domain-specific models that can assist in interpreting complex clinical documentation. Future work will focus on real-world validation and expanding the model to other procedural report types.

Colorectal cancer (CRC) remains a leading cause of cancer-related morbidity and mortality worldwide.^1^ Early detection through regular screening, particularly colonoscopy, has proved effective in reducing incidence and improving survival rates.^2^ However, ensuring timely and appropriate follow-up care is crucial to maximizing the benefits of screening programs. Colonoscopy reports serve as essential documents that guide clinical decision-making, yet inconsistencies and incomplete documentation often hinder their effectiveness.^3^ Studies have shown that a relevant percentage of colonoscopy reports lack clear follow-up recommendations, which can lead to missed opportunities for timely surveillance and increased risk of CRC progression.^4^ The manual processing and extraction of information from clinical notes, including colonoscopy reports, present substantial challenges. Clinicians often spend a considerable portion of their time on documentation tasks, contributing to burnout and detracting from patient care. This administrative burden is not only taxing for health care providers but also impacts the quality and timeliness of patient care.^5^ Advancements in artificial intelligence (AI), particularly the application of large language models (LLMs), offer promising solutions to these challenges. LLMs have reported proficiency in processing and interpreting clinical text, enabling the extraction of pertinent information and standardization of documentation. By automating the analysis of colonoscopy reports, LLMs can facilitate the generation of personalized follow-up recommendations, ensuring adherence to clinical guidelines and enhancing patient outcomes.^6^^,^^7^

This project explores the integration of LLM-based clinical natural language processing (NLP) methods to analyze colonoscopy and pathology reports, with the goal of improving the extraction of key clinical details that inform follow-up decision-making. By streamlining documentation processes and standardizing the capture of clinically relevant information, we aim to reduce clinician workload, mitigate burnout, and enhance the quality of care in CRC screening workflows. Despite their strong performance, large language models come with relevant limitations, particularly regarding scalability and deployment in real-world clinical settings. These models are often computationally intensive, requiring substantial hardware resources and cloud infrastructure to operate efficiently, conditions that may not be available in many health care environments. In addition, the size and complexity of these models can raise concerns around latency, cost, and integration into clinical workflows that demand real-time or near-real-time responses. To address these challenges, this project applies knowledge distillation, a technique that transfers knowledge from a large, high-performing model to a smaller, more efficient student model. This approach enables the development of smaller models that maintain task-specific performance while considerably reducing computational requirements. Such models are more practical for integration into clinical systems and better suited for large-scale deployment across institutions. In the context of gastroenterology, where timely review of procedural and diagnostic reports is essential, scalable and efficient NLP tools can help close workflow gaps without introducing technological burden.

## Methods

### IRB Approval Statement

This study was reviewed and approved by the Mayo Clinic institutional review board (Protocol Number: 24-004310). The requirement for written informed consent was waived by the institutional review board.

### Dataset Description

We used a substantial dataset comprising 5500 colonoscopy reports and 7000 pathology reports, all extracted from the Mayo Clinic’s enterprise data warehouse. This warehouse is built on Google BigQuery,^8^ enabling efficient querying and handling of large-scale clinical data. The reports spanned a 5-month period, from January 1, 2024, to June 30, 2024, providing a rich and diverse dataset. This dataset served as the foundation for both the fine-tuning and knowledge distillation processes. The volume and specificity of the data were critical in training a model that could generalize well within the domain of gastroenterology-focused medical report summarization and information extraction. The inclusion of both colonoscopy and pathology reports also allowed the model to learn from multiple perspectives of the clinical workflow, thereby enhancing its ability to capture clinically relevant patterns and language structures.

The colonoscopy reports used in this project contained general information on the surgery. These reports include details such as the number, size, and removal method of the patient's polyps. They also documented where the polyps were in the colon, what the patients' prep score was and if the colonoscopy was complete or not. This information is useful to the endoscopists in telling the patient when they should return for their next follow-up exam. The pathology reports used in this project correspond to polyp specimens removed during colonoscopy procedures. These reports can contain a wide range of diagnostic classifications; however, the primary objective of our model was to classify each polyp into one of four predefined categories based on histological features. These categories were selected for their clinical relevance. Certain polyp types are associated with a higher risk of malignant transformation and therefore require closer follow-up.

The 4 categories used for classification were: tubular adenomas, hyperplastic polyps, sessile-serrated polyps, and traditional-serrated polyps. By focusing on these categories, the model supports standardized follow-up interval recommendations and contributes to more efficient risk stratification in colorectal cancer screening.^9^ It is important to note that the colonoscopy and pathology reports in this dataset were not linked at the patient or procedure level. As a result, the 2 report types were treated as independent samples during model training and evaluation. This also explains the difference in sample sizes between the reports. The model was designed to process each report individually, without attempting to integrate or infer relationships between paired reports.

### Dataset Preprocessing

The datasets used in this project consisted of medical reports written by clinicians, many of which followed a semistructured or templated format. Minimal preprocessing was performed to ensure the text was clean and standardized for use in natural language processing tasks. Specifically, we removed uneven white spaces, including extra line breaks and irregular spacing between words or sections. This step was necessary to improve text readability for the model. Tokenization, stop word removal, and stemming were not applied, as preserving the domain-specific language in medical reports is considered important for maintaining model accuracy.^10^ The dataset was then divided into training and testing subsets with 80% for training and 20% for testing. All processing was done on the VertexAI^11^ framework made available through Google Cloud. Vertex AI was instrumental in model tuning, distillation, and deployment, providing a robust platform for developing our distilled model. For data manipulation and preprocessing, we extensively used the Pandas library, which facilitated efficient handling of the large datasets involved.^12^

Table 1 presents the demographic characteristics of the dataset, limited to age and sex. These were the only demographic variables consistently available within the colonoscopy and pathology reports used for this study. Other characteristics, such as race and ethnicity, were not included because they were either unavailable in the source text or inconsistently documented. Although race and ethnicity may be relevant in some areas of medical research, current clinical guidelines and literature indicate that age and sex are the primary biological factors influencing colorectal polyp development, surveillance intervals, and overall colonoscopy outcomes.^7^ Therefore, the available demographic information was considered appropriate for the scope and clinical focus of this project.

**Table 1:** Demographic Characteristics of Colonoscopy and Pathology Report Cohorts

Characteristics	Colonoscopy Reports (n=5500)	Pathology Reports (n=7000)
Sex		
Male	2668 (48.5%)	3467 (49.5%)
Female	2832 (51.5%)	3533 (50.5%)
Age group (y)		
40-49	321 (5.8%)	551 (7.9%)
50-59	1496 (27.2%)	1766 (25.2%)
60-69	1657 (30.1%)	2183 (31.2%)
70-79	1766 (32.1%)	2137 (30.5%)
80-89	224 (4.1%)	312 (4.5%)
≥ 90	36 (0.7%)	51 (0.7%)

### Reference Standard and Ground Truth Labeling

Ground truth labels for polyp classification were derived directly from the final diagnostic interpretations documented in the pathology reports. These reports were generated in routine clinical practice at Mayo Clinic and served as the reference standard for model development and evaluation.

For the purposes of this study, pathology diagnoses were mapped to 1 of 4 predefined histologic categories: tubular adenomas, hyperplastic polyps, sessile serrated polyps, and traditional serrated polyps. Mapping was performed using the diagnostic terminology contained in the pathology report text.

Because the ground truth labels were based on clinically generated pathology interpretations rather than an independent research annotation workflow, no separate manual annotation of the full dataset was performed, and inter-annotator agreement was not formally assessed.

### Knowledge Distillation

Knowledge distillation is a model compression technique in which a smaller, more efficient model (the student) is trained to approximate the behavior of a larger, more complex model (the teacher).^13^ Instead of learning solely from the output labels, the student learns from the soft labels, probability distributions over output classes, produced by the teacher model. This allows the student to capture not only correct answers, but also the confidence and nuanced decision boundaries of the teacher. The result is a smaller model that was designed to preserve task-specific performance while reducing computational requirements relative to the teacher model.

In this project, we used PaLM 2 Text Unicorn,^14^ an LLMtrained on ∼340 billion parameters, as our teacher model.^15^ Its high capacity and broad generalization made it well-suited for extracting and summarizing complex clinical text. Using Vertex AI’s tuning and distillation tools, we trained a smaller student model with ∼130 billion parameters. The distilled student model was specifically trained on 2 key clinical NLP tasks: analyzing colonoscopy reports and extracting diagnostic classifications from pathology reports. It was designed to preserve task-specific performance within these domains while improving inference speed relative to the teacher model.

The distillation pipeline consisted of teacher inference on a curated training set, supervised tuning of the student model on teacher-generated targets, and validation on a held-out set. For each training example, the teacher model received raw report text along with task-specific instructions and produced a probability distribution over candidate classifications or task-specific outputs. These teacher-generated outputs served as the targets for student-model training.

The student model was trained using a distillation-based procedure, with a combined loss of Kullback-Leibler Divergence Loss^16^ and Cross-Entropy Loss. Training was optimized for 2 task formulations: classification of pathology findings and structured extraction/interpretation of colonoscopy report content.

To improve consistency, prompting of the teacher model followed a template-based strategy in which we ensured every prompt for the teacher model followed the same structure. Prompts were iteratively refined to improve output completeness, format adherence, or clinical specificity. The resulting student model reported faster inference than the teacher model, although its size may still present practical constraints for deployment in some clinical environments.

Operational benchmarking in this study was limited to relative inference speed comparison between the student and teacher models under the same evaluation conditions.

### Statistical Analyses

Model performance was summarized using accuracy, precision, recall, and F1 score. Ninety-five percent confidence intervals were calculated for primary performance metrics on the held-out test set using bootstrapping.

## Results

The project spanned several phases. The initial phase focused on setting up and fine-tuning a large pre-trained language model. Subsequently, knowledge distillation was employed to develop the distilled model. During this phase, important efforts were made to ensure the distilled model retained the performance of the teacher model. These efforts included adjusting the prompt given to the model and changing the parameters. The final evaluation phase assessed both accuracy and efficiency. Relative to the teacher model, the distilled student model showed a 31.5% improvement in inference speed under our evaluation conditions. We tested the model with six clinician-defined questions for polyp categorization: (1) how the polyps were removed; (2) the number of polyps; (3) the size of each polyp; (4) the histologic classification of each polyp; (5) the location of the polyps; and (6) whether any polyps were cancerous. These questions were specified by a board-certified gastroenterologist based on information commonly used to determine follow-up intervals. Three gastroenterology specialists reviewed the model’s outputs and independently recorded their own answers, indicating agreement or disagreement with the model for each item. Although evaluation focused on six clinician-defined questions, the system supports broader free-text queries.

**Figure 1:**
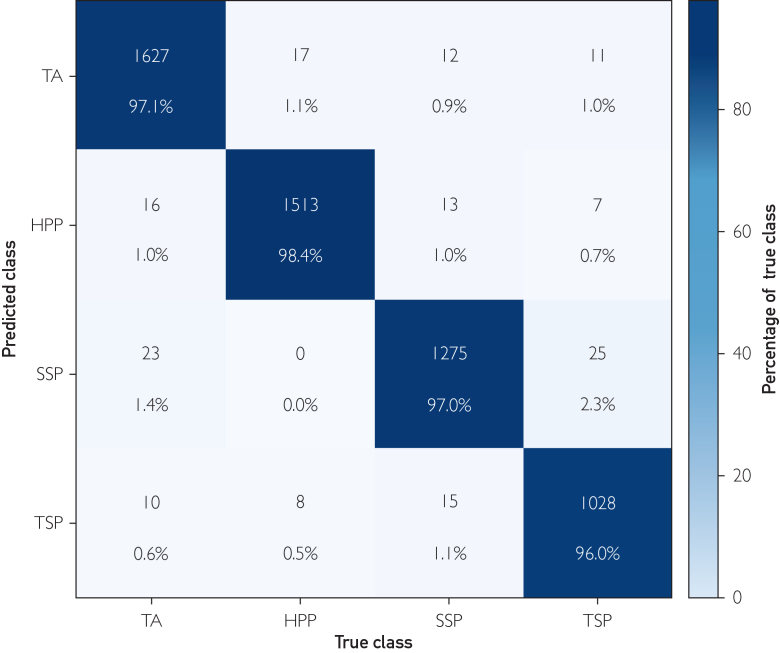
Normalized confusion matrix for polyp classification (training set) of the distilled model. Each cell shows the percentage of true class instances predicted as each class. TA, tubular adenomas; HPP, hyperplastic polyps; SSP, sessile serrated polyps; TSP, traditional serrated polyps.

Table 2 summarizes performance across the evaluated models in this study on the testing set. The accuracies seen in the table are on the ability to classify polyps correctly and answer questions based on the reports. Within the comparator set, the distilled model achieved the highest overall accuracy with a 95% confidence interval of 93.9%-96.5%, precision, recall, and F1 score. To further analyze model performance, we generated a confusion matrix based on the training dataset. This matrix in Figure 1 reflects the distilled model’s classification accuracy across the 4 polyp categories at the final epoch of training, providing insight into how well the model learned from the data it was exposed to while highlighting any consistent misclassifications. For polyp classification, the model achieved high concordance with the ground truth, with a normalized confusion matrix showing correct predictions exceeding 96% for all four histologic categories. Class-wise precision ranged from 0.96 to 0.98, recall from 0.96 to 0.98, and F1 scores from 0.96 to 0.98, indicating balanced sensitivity and specificity across categories. Errors were minimal and largely limited to clinically adjacent classes, such as sessile serrated polyps and traditional serrated polyps. Overall, the model’s high accuracy, consistent performance across categories, and low misclassification rates highlight its reliability for extracting structured diagnostic information from gastroenterology reports.

**Table 2:** Model Performance Comparison (Testing)

Model Version	Accuracy (95% CI)	Precision	Recall	F1 Score
PaLM 2 Text Bison	89.6 (88.8%-90.4%)	0.85	0.84	0.84
Gemini 1.5 Flash	93.4 (91.7%-95.1%)	0.89	0.88	0.88
Distilled Model	95.2 (93.9%-96.5%)	0.95	0.97	0.97

**Figure 2:**
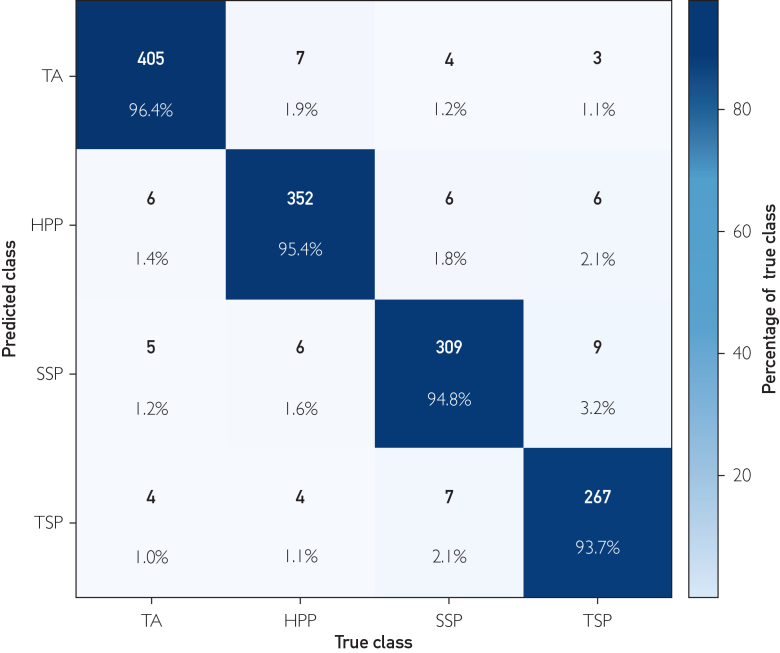
Normalized confusion matrix for polyp classification (testing set) of the distilled model. Each cell shows the percentage of true class instances predicted as each class. TA, tubular adenomas; HPP, hyperplastic polyps; SSP, sessile serrated polyps; TSP, traditional serrated polyps.

On the held-out testing set, the distilled model maintained high and balanced performance across all four polyp classes. The normalized confusion matrix in Figure 2 showed correct predictions in approximately 94%-97% of cases for each histologic category, with misclassifications distributed sparsely across off-diagonal cells. Class-wise precision ranged from 0.94 to 0.96 and recall from 0.94 to 0.97, yielding F1 scores between 0.94 and 0.97 and specificities around 0.98 for all classes. These results indicate that the model generalizes well to the held-out test set and reliably distinguishes between tubular adenomas, hyperplastic polyps, sessile serrated polyps, and traditional serrated polyps.

## Discussion

The implementation of knowledge distillation in this project proved to be an effective strategy for developing a smaller, domain-specific model capable of processing colonoscopy and pathology reports. By distilling knowledge from a larger base model into a smaller, task-focused version, we were able to retain strong performance on key clinical NLP tasks while also improving inference speed. In our evaluation, the student model reported a 31.5% improvement in inference speed relative to the teacher model. These findings suggest that knowledge distillation may improve the practicality of domain-specific NLP systems, although the student model still has substantial computational requirements.

One of the most important findings was the model’s strong performance within its domain training. This confirms the value of domain-specific tuning, especially in health care contexts where clinical language can be highly specialized. However, the model’s ability to generalize beyond its training data was notably limited. When tested on tasks or input types outside the colonoscopy and pathology report domain; performance dropped. This limitation underscores a well-known challenge in medical AI: the trade-off between specificity and generalizability.^17^ The effectiveness of our approach is closely tied to the quality, diversity, and domain alignment of the training dataset. Expanding this dataset to include a broader range of clinical documentation could improve the model’s robustness and applicability.

Another key consideration is the static nature of our model. Unlike commercial large language models such as GPT-4^18^ or Gemini Flash 1.5,^19^ which are frequently updated and may experience changes in performance or behavior over time, our distilled model is version-controlled and static. This ensures consistent behavior across deployments, which is particularly important in clinical environments that demand reproducibility, auditability, and regulatory compliance. A static model can be validated, integrated, and maintained with confidence, reducing uncertainty and supporting long-term use in health systems.^20^ This characteristic is especially valuable in the context of gastroenterology, where accurate and repeatable interpretation of procedural reports can directly impact patient care.

Despite these promising outcomes, several limitations must be acknowledged. The model’s performance is inherently dependent on the representativeness and quality of the training data. Additionally, although the student model is smaller than the teacher, it still has substantial computational requirements. This study also did not include formal benchmarking of throughput, memory usage, or computational cost; therefore, efficiency conclusions should be interpreted primarily in terms of relative inference speed. Moreover, our evaluation focused primarily on colonoscopy-related tasks; thus, the ability of the model to generalize to other areas of medicine, such as cardiology or dermatology, remains untested. Another limitation is that baseline comparisons were limited to general-purpose LLMs and did not include established clinical NLP models or rule-based systems. Therefore, the practical advantages of the distilled model should be interpreted relative to the selected comparator models rather than as evidence of superiority over the broader landscape of clinical NLP approaches. Future work should include head-to-head comparisons with established clinical NLP models and rule-based systems to better define the relative advantages, tradeoffs, and implementation value of distilled domain-specific LLMs in gastroenterology workflows. In addition, future work could include expanding the dataset to incorporate a wider range of medical report types and exploring methods, such as multi-task learning or transfer learning, to enhance generalization across domains.

Looking forward, this approach has the potential to extend beyond colonoscopies. Applying knowledge distillation techniques to other subfields of medical NLP, such as radiology or broader pathology reports, may help scale efficient AI tools across different specialties. Additionally, investigating how distilled models can integrate with electronic health records systems or clinical decision support tools would be an important step toward real-world clinical adoption.

## Conclusion

This project addresses a critical gap in gastroenterology: the time-consuming and inconsistent process of manually reviewing colonoscopy and pathology reports to determine appropriate follow-up care. Incomplete or unclear documentation in these reports can lead to missed surveillance opportunities and increased risk for CRC progression. To mitigate this, we developed a domain-specific LLM-based NLP model using knowledge distillation to streamline the interpretation of procedural and diagnostic text.

Our approach resulted in a smaller, faster model that retains strong task performance while improving inference speed relative to the teacher model. By focusing on a specific domain and leveraging a high-quality dataset, we ensured the model could extract key clinical details and assist in generation of guideline-based follow-up recommendations.

Of importance, our model is designed as a static, version-controlled tool, offering consistent and reproducible performance, which is a distinct advantage over commercial large language models that evolve over time and may lack transparency or auditability. Overall, this work reports the feasibility and clinical relevance of applying knowledge distillation in health care AI. It contributes to ongoing efforts to reduce documentation burden, support clinical decision-making, and improve the quality of CRC screening workflows. Future work will focus on expanding this approach to other types of clinical reports and validation of its performance in real-world health care systems.

## Potential Competing Interest

Given his role as Editorial Board Member, Dr Chaudhry, had no involvement in the peer-review of this article and has no access to information regarding its peer-review. The remaining authors disclose no conflict of interest.

## Ethics Statement

This study was reviewed and approved by the Mayo Clinic institutional review board (Protocol Number: 24-004310). The requirement for written informed consent was waived by the institutional review board.
